# Supplementary material for: Nonequilibrium continuous phase transition in colloidal gelation with short-range attraction
Source: Nat Commun. 2020 Jul 16;11:3558. doi: 10.1038/s41467-020-17353-8 (PMC7367344; doi:10.1038/s41467-020-17353-8)
Supplement: Supplementary file 1 — Supplementary Information [file 41467_2020_17353_MOESM1_ESM.pdf]

Supplementary Information for article
"Nonequilibrium continuous phase transition in colloidal gelation with short-range attraction"

Rouwhorst *et al.*

SUPPLEMENTARY NOTE 1: GELATION STATE DIAGRAM

To put our system in the context of previously studied gelation, we compute the Baxter temperature τ as outlined in [1]. This allows us in particular to compare our state points with those of the adhesive hard sphere system, which should map onto our short-range attractive system following the Noro-Frenkel correspondence [2]. The reduced temperature τ is related to the range and depth of the attractive potential according to Eq. 1 of Ref [1]. Computing this parameter for our system, and plotting the mean coordination number as a function of τ, we find that the onset of gelation for the volume fraction $\phi = 0.12$ presented in the manuscript occurs at $\tau \sim 0.1$, as shown in Supplementary Figure 1a. Similarly, we determined the onset of gelation in a broader range of volume fractions (ϕ = 0.03, 0.06, 0.12 and 0.24) in the Mie-potential simulations. The resulting state points are indicated in the model adhesive hard-sphere diagram in Supplementary Figure 1b. For each investigated volume fraction, the fluid state is indicated by black triangles, and the gelled state by red dots. For $\phi = 0.12$ investigated in the manuscript, the gelation transition is located slightly below the binodal, in good agreement with previous work on adhesive hard spheres [1]. Towards lower volume fractions, the fluid-gel boundary drops significantly, in qualitative agreement with [1], but the drop occurs more steeply. The difference may be partially explained by the different definition of the gelation onset, based on a constant autocorrelation function measured by dynamic light scattering in [1], and based on the percolation of the largest cluster in the present work. We also note that the indicated fluid-fluid binodal in Supplementary Figure 1 is known to become metastable with respect to the solid-liquid binodal (not shown) when the range of attraction is shorter than 0.14r. This is close to the investigated attraction range of the critical Casimir interaction studied; however, we do not observe any sign of solid-liquid transition in the range of attractions we investigated.

The relation to the fluid-fluid boundary is best shown in Supplementary Figure 2, where we indicate the onset of gelation in the phase diagram of simulated adhesive hard spheres [3]. The gelation onset is located below the phase boundary, and drops steeply towards lower volume fraction. As pointed out in [1], this is different from colloidal depletion systems, for which the gelation onset occurs at the fluid-fluid phase boundary [4]. The gelation of our system thus compares with that of adhesive hard spheres, and is also consistent with the gelation line in a recent paper on particles with short-range interacting random patches [5].

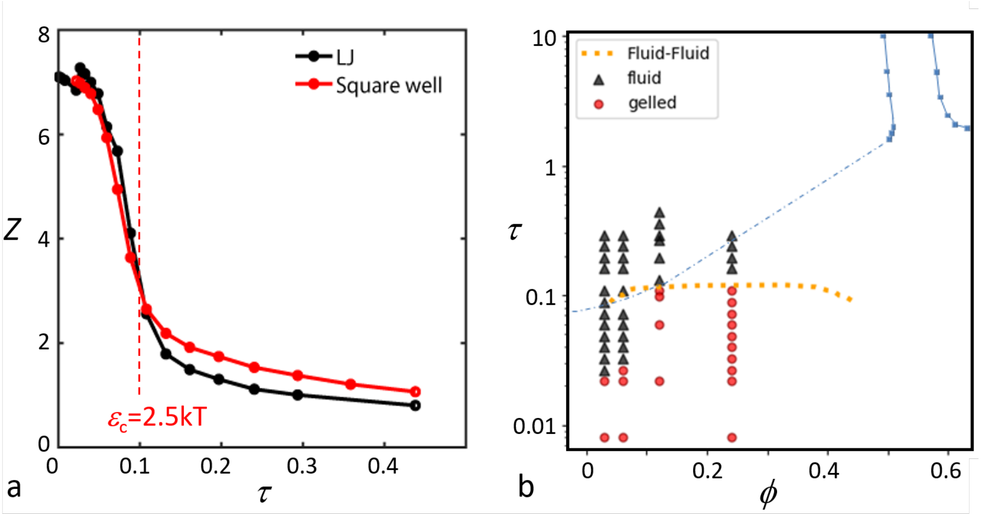

Supplementary Figure 1. Gelation transition in the Baxter diagram for adhesive hard spheres (AHS). (a) Mean coordination number as a function of the Baxter parameter τ for the simulation with volume fraction $\phi = 0.12$ studied in the main manuscript. Gelation occurs at $\epsilon_c = 2.5kT$, corresponding to $\tau \sim 0.1$. (b) Fluid and gel state points at particle volume fractions $\phi = 0.03, 0.06, 0.12$ and 0.24 of the simulations, indicated in the phase diagram of the AHS system. Fluid states are indicated by black triangles, and gel states by red dots. Towards low volume fractions, the gel line decreases significantly below the fluid-fluid coexistence boundary, in qualitative agreement with previous results on nanoparticles with short-range attraction (dashed line) [1].

SUPPLEMENTARY NOTE 2: SCALING IN A WIDER RANGE OF VOLUME FRACTIONS

In the main manuscript, we have shown that gelation manifests as a nonequilibrium percolation transition. We observed that as a function of the mean coordination number, the largest cluster diverged. Here, we provide more evidence by exploring the percolation behavior in a wider range of volume fractions, and for a very different experimental system, namely protein microparticles, exhibiting short-range attractive interactions very different from those of the colloidal model system in the manuscript. Details of these protein microparticles, for which gelation was induced by slow acidificaton, are described below. For these different systems, the fraction f_z of particles in the largest cluster as a function of average coordination number is shown in Supplementary Figure 3a. The data reveals divergence at a system and volume-fraction specific critical coordination number z_c, in a way

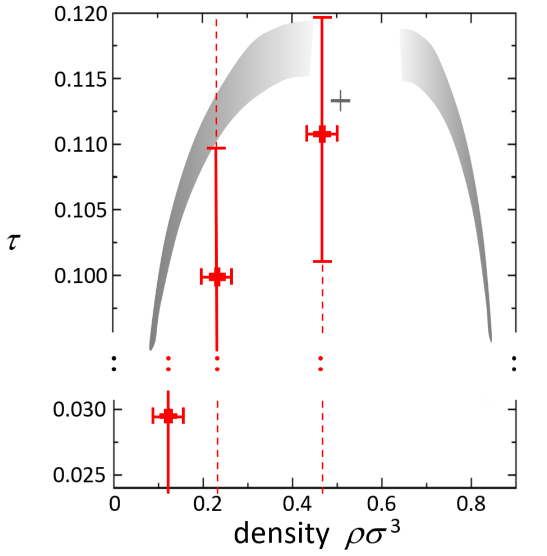

Supplementary Figure 2. Gelation onset with respect to fluid-fluid phase boundary. Red crosses and error bars indicate the onset of gelation in the Mie-potential simulations at volume fractions 0.06, 0.12 and 0.24 (densities $\rho\sigma^3 = 0.12$, 0.24 and 0.48, respectively), compared to the fluid-fluid boundary as determined in Monte Carlo simulations for adhesive hard spheres according to [3] (gray areas, representing the range of phase boundaries found for different system sizes). The onset of gelation lies below the fluid-fluid boundary, and decreases sharply towards lower volume fractions (note the break in the y-axis), to the left side of the critical point (estimated location indicated by the black cross at $\tau = 0.1133$, $\rho = 0.508$). Error bars indicate standard deviation in the determination of the gelation boundary.

similar to the data in the manuscript. When scaling the x-axis by the system-specific z_c, all curves collapse in their approach of z_c, see Supplementary Figure 3b. Furthermore, when we investigate the scaling of f_z upon approaching z_c by plotting f_z as a function of $z_c - z$, all data overlap and exhibit the same divergence with the exponent -1.6 (blue dotted line), identical to the scaling reported in the manuscript. This remarkable collapse for the different systems and volume fractions suggests that there is a general mechanism underlying the gelation in all these systems.

We note that f_z after gelation does not necessarily collapse: depending on the volume fraction (and attraction), the largest cluster may contain different fractions of particles. This is clearly shown in two reconstructions at different volume fractions of the simulated

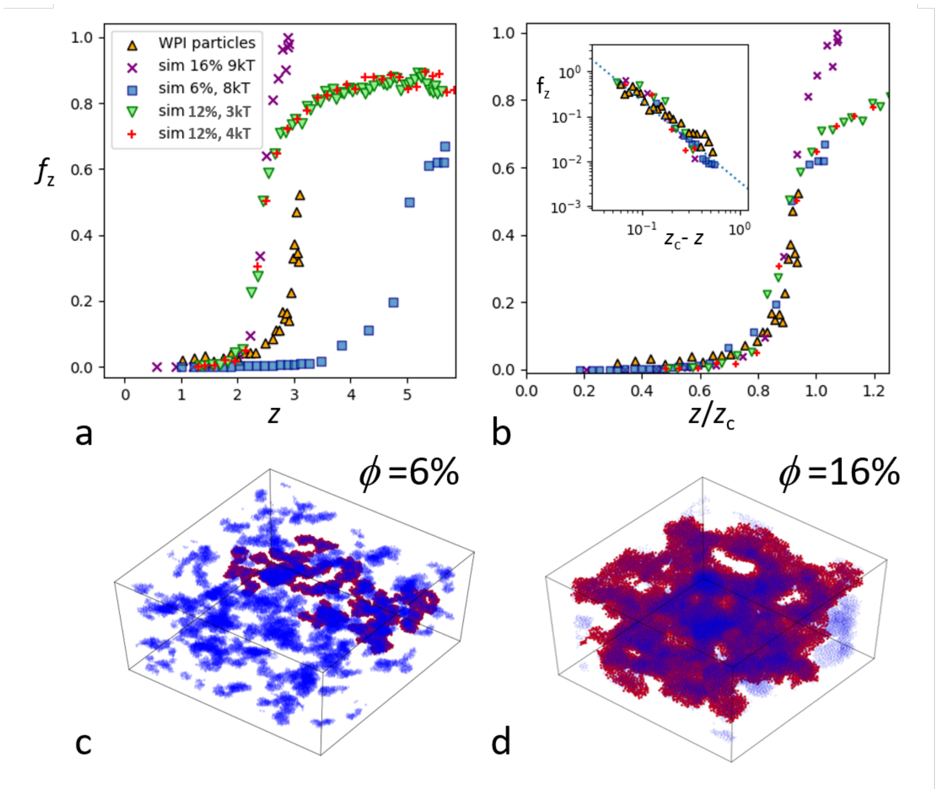

Supplementary Figure 3. Percolation at various volume fractions and in different systems. (a) Fraction of particles in the largest cluster, f_z, as a function of the total mean coordination number, z, in simulations for particle volume fractions $\phi = 0.06$, 0.12 and 0.16, and in an experimental system of whey protein isolate (WPI) microparticles (see legend). (b) Same data of f_z as a function of normalized coordination number z/z_c. Inset: Divergence of f_z upon approaching the critical coordination number z_c. (c,d) Reconstructions of the late-stage (gelled) system, highlighting the largest, percolating cluster (red particles) coexisting with the smaller particle clusters (transparent blue) for particle volume fractions of 6% (c) and 16% (d).

system after gelation in Supplementary Figure 3c and d. Red particles indicate the largest (space-spanning) cluster, and faint blue particles indicate all other clusters. While both red-particle clusters percolate the field of view, the one for the higher volume fraction contains an even larger fraction of particles, making it dominate the field of view.

Finally, we note that also the cluster mass distributions for all investigated systems and volume fractions are robust and exhibit the same power-law distribution as those presented in the manuscript. As an example, we show the distribution of cluster masses before gelation in Supplementary Figure 4. The figure shows data for the two experimental systems (critical

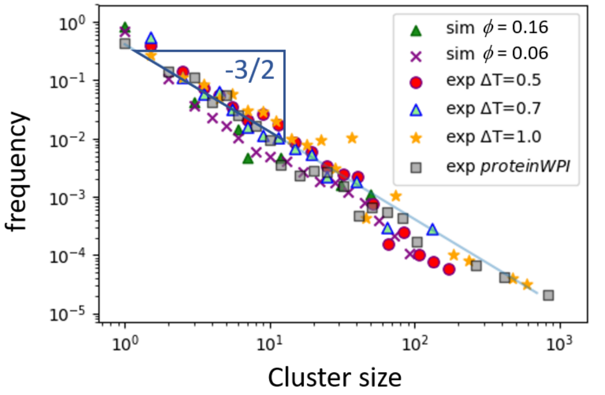

Supplementary Figure 4. Cluster mass distributions before gelation.

Cluster mass distributions for critical Casimir experiments at various ΔT, experiments on protein microparticles, and Mie-potential simulations at two different volume fractions at aggregation stages just before gelation. All data closely follow power-law distributions with slope -3/2 (light blue line).

Casimir colloidal system at different ΔT, i.e. different attractive strength, and protein microparticles), and the simulations at two different volume fractions (0.16 and 0.06). Despite the different z_c and different cluster appearance after gelation (cf. Supplementary Figure 3c and d), the cluster mass distribution remains robust, being again described by a power law with slope $-3/2$. These results support our conclusions in the manuscript and indicate some generality.

SUPPLEMENTARY NOTE 3: PROTEIN MICROPARTICLES

We briefly outline some details of the protein microparticle system here. These particles with an average diameter of $1.9\mu m$ and a polydispersity of 15% are made by preclustering whey proteins using the double emulsification method as described in [6]. To achieve cold-set gelation of the particles, the pre-clustering was accompanied by a mild heat treatment to $\sim 80°C$, close to the denaturation temperature of the proteins [7]. The particles were size-selected down to 15% polydispersity by repeated centrifugation. Sugar was added to the final aqueous solution to match the density and refractive index of the microparticles to that of the solvent. Subsequently, acid-induced gelation was achieved by adding 0.36% by weight of glucono δ-lactone (GDL), lowering the pH and adjusting it towards the isoelectric point of

the proteins. This causes a reduction of the electrostatic repulsion of the protein particles, thereby rendering them unstable and inducing aggregation. We estimate the interaction potential in the framework of DLVO theory as a function of the zeta potential, taking into account the charge density of the proteins and the ion density in the solvent. Using these estimates, we find that close to gelation the interaction energy of protein microparticles is $\sim 30k_{\mathrm{B}}T$, resulting in particle attachment with very low probability of detachment or rearrangement. The resulting approach to gelation is nevertheless very similar to both our critical Casimir colloidal model system and the simulations (both Mie and square-well potential), as shown in Supplementary Figures 3 and 4.

SUPPLEMENTARY REFERENCES

[1] A. P. R. Eberle, N. J. Wagner and Ramon Castaneda-Priego, Dynamical arrest transition in nanoparticle dispersions with short-range interaction Phys. Rev. Lett. 106, 105704 (2011).

[2] M. G. Noro and D. Frenkel, Extended corresponding-states behavior for particles with variable range attractions. J. Chem. Phys. 113, 2941 (2000).

[3] Mark A. Miller and Daan Frenkel, Competition of percolation and phase separation in a fluid of adhesive hard spheres. Phys. Rev. Lett. 90, 135702 (2003).

[4] P. J. Lu, E. Zaccarelli, F. Ciulla, A. B. Schofield, F. Sciortino, and David A. Weitz, Gelation of particles with short-range attraction. Nature 499, 453 (2008).

[5] G. Wang and J. W. Swan, Surface heterogeneity affects percolation and gelation of colloids: dynamic simulations with random patchy spheres. Soft Matter 15, 5094 (2019).

[6] Saglam, D., Venema, P., de Vries, R., Sagis, L. M. C. and van der Linden, E. Preparation of high protein micro-particles using two-step emulsification. Food Hydrocoll. 25, 1139–1148 (2011).

[7] C. J. L. van Balen, *Colloidal gelation of proteins in the light of universal criticality*, Master Thesis Wageningen University and University of Amsterdam (2019).
