# Supplementary material for: Nonequilibrium continuous phase transition in colloidal gelation with short-range attraction
Source: Nat Commun. 2020 Jul 16;11:3558. doi: 10.1038/s41467-020-17353-8 (PMC7367344; doi:10.1038/s41467-020-17353-8)
Supplement: Supplementary file 3 — Description of Additional Supplementary Files [file 41467_2020_17353_MOESM3_ESM.pdf]

Description of Additional Supplementary Files

Supplementary Movie 1: Animation of the simulated colloidal gelation

Animation of the gelation process for a simulated colloidal system at volume fraction $\phi=$ 0.16. Shown is the evolution over 300 time units following an instantaneous change in the particle-particle attraction strength from $1 k_B T$ to $4\ k_B T$. The colour scale represents the particle-particle coordination: black particles have 0 neighbours within the attractive range of the potential; yellow ones have 12 neighbours. Low coordination particles at cluster periphery are seen to detach and re-attach frequently, while high-coordination particles at the centre are much less mobile.
