# Supplementary figures and images for: Nonequilibrium continuous phase transition in colloidal gelation with short-range attraction
Source: Nat Commun. 2020 Jul 16;11:3558. doi: 10.1038/s41467-020-17353-8 (PMC7367344; doi:10.1038/s41467-020-17353-8)

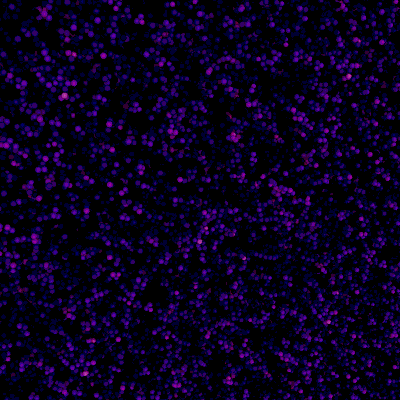

Supplement: Supplementary file 4 — Supplementary Movie 1 [file 41467_2020_17353_MOESM4_ESM.gif]
